# Effect of Different Grain for Green Approaches on Soil Bacterial Community in a Karst Region

**DOI:** 10.3389/fmicb.2020.577242

**Published:** 2020-10-30

**Authors:** Huijun Chen, Wanxia Peng, Hu Du, Tongqing Song, Fuping Zeng, Feng Wang

**Affiliations:** ^1^Key Laboratory of Agro-ecological Processes in Subtropical Region, Institute of Subtropical Agriculture, Chinese Academy of Sciences, Changsha, China; ^2^Huanjiang Observation and Research Station of Karst Ecosystem, Chinese Academy of Sciences, Huanjiang, China

**Keywords:** soil properties, soil bacterial community, revegetation, karst ecosystem, illumina sequencing

## Abstract

Soil bacteria participate in nutrient cycling above and below ground to promote ecosystem stability and health. However, the relationship of soil bacteria and environmental factors following the Grain for Green (GfG) program remains poorly understood in southwest China. Soil samples were collected from seven Grain for Green sites that had been revegetated for 15 years. Four of these sites were afforested with a different tree species: *Zenia insignis* (ZI), *Toona sinensis* (TS), *Castanea mollissima* (CM), and *Citrus reticulate* (CR). One site was revegetated with *Zenia insignis* and Guimu-1 elephant grass (ZG), and one with only Guimu-1 elephant grass (GM). The remaining site, abandoned cropland (AC), was left to regenerate naturally. Here, we used Illumina sequencing of 16S rRNA genes to explore how the Grain for Green project affected soil bacterial community. We found that Actinobacteria, Proteobacteria, Chloroflexi, and Acidobacteria were the dominant phyla in these soils. The dominant genera at each revegetation site were also different. The CM, ZI, TS, and AC sites were dominated by Micromonospora, ZG was dominated by Streptomyces, and CR and GM were dominated by Subgroup 6. The bacterial structure was most similar in AC and TS. Correlation analysis showed that the ratio of C:P had positive effects on KD4-96, Intrasporangiaceae, and *Gaiella*. The ratio of soil N:P was significantly positively correlated with *Cupriavidus* and *Kribbella*. The combination of planting *Zenia insignis* and Guimu-1 elephant grass had the best edaphic benefits, and the approach of planting *Citrus reticulate* and *Toona sinensis* needs to be improved. Redundancy analysis (RDA) revealed that plant Simpson index, and soil N:P contributed to 16 and 15.7% of the total variations in the soil bacterial community composition, respectively. Our results suggested that plant diversity (Simpson index) and soil stoichiometric ratio (N:P) were the important factors affecting the bacterial community, and phosphorus was the limiting factor of the bacterial community in the Grain for Green karst region. In the future, revegetation should be accompanied with phosphorus fertilizer and polycultures should be considered.

## Introduction

Bacteria are the most abundant microbes in soil and are crucial for ecosystem stability and nutrient cycling, especially carbon and nitrogen (Pan et al., 2014; Eo and Park, 2016; Zeng et al., 2017). Although bacteria are tiny, their function for soil could promote and keep biodiversity by driving and regulating ecosystem processes (Herzog et al., 2015). Furthermore, bacteria are sensitive to minor changes in ecosystems and can be regarded as an indicator of soil quality, and their structure and diversity are determined by their external environment (Sui et al., 2019). It has been reported that microbial communities were not stochastically distributed on a wide scale and their biogeographical patterns were more influenced by soil type and land use (Ranjard et al., 2010). Recently, many anthropogenic activities were carried out to promote agricultural production and ecological restoration (Turley et al., 2019; Bonner et al., 2020; Tian et al., 2020). The Grain for Green program (GfG), a reforestation and ecological restoration program, changed local edaphic characteristics by shaping the structure of bacterial community (Ren et al., 2017; Zhao et al., 2019). Due to the wide geographic implementation of the GfG project, the effect of the project has not been evaluated at many local sites.

The GfG program, which was initiated by China to alleviate poverty and hasten rural development, has changed soil properties notably. For example, Shao et al. (2019) found that afforestation changed soil properties and affected soil quality (Zhang et al., 2019). The GfG also promoted soil carbon and nitrogen accumulation in secondary forests in the subtropical karst region, which were relative to soil depth (Hu et al., 2018). Afforestation might affect the soil bacterial community by changing soil properties (Zhao et al., 2019), while the soil environment determines soil bacteria community composition and structure directly (Zhao et al., 2019; Zhang et al., 2020). Due to quite distinctive physiographies, climates, and ecosystems in the different regions, the universal revegetation method was not applicable. When the GfG was set to end in 2017, knowledge of the ecological impacts of the program on the soil environment was urgently needed to determine the best implementation of the revegetation methods used in the GfG program. Therefore, it is important to explore the internal relationships between the soil bacterial community and external environment under different revegetation practices at local scales.

The karst region of southwestern China, one of the largest continuous karsts in the world, is known for its unique landscapes and rich biodiversity (Wang et al., 2019). The increasing human population has driven land overuse and deforestation, and it has intensified poverty (Peng et al., 2008). Reforestation has been widely adopted to restore soil fertility and ecosystem services in the rocky desertified region of southwestern China (Hu et al., 2016). Previous studies have investigated the relationships between vegetational and topographic factors (Du et al., 2013; Yu et al., 2014; Han, 2017). It was also found that agricultural abandonment could recover soil aggregation in this karst region (Liu et al., 2020). Furthermore, Song et.al (2018) and Peng et al. (2019) explored the driver of the spatial distribution of the main microbial taxa and soil microbial richness and diversity in a 25-ha karst broadleaf forest in southwest China, respectively. However, few studies focused on the bacteria community and environmental factors under several different vegetation restoration practices. Here, we conducted our search at Guzhou Village, Guangxi Province, China. The overall goals of our study were: (1) to explore how the soil bacterial community and environmental factors vary among the different sites; and (2) to determine the relationship between soil bacteria and environmental factors. For the same cropland substrate, different revegetation strategies (such as managed and natural vegetation restoration) may have different ecological benefits, which makes a comparison among each strategy necessary. Our findings could be used to guide the future selection and combination of species for the revegetation of degraded karst regions in southwest China.

## Materials and Methods

### Study Area and Sampling

Our study was conducted at Guzhou Village (24°50’N, 107°55’E), Huanjiang County, Guangxi Province, China, which has typical karst ecosystems. This region has a subtropical monsoon climate with a mean annual precipitation of 1,389 mm and a mean annual temperature between 16.5°C and 20.5°C. The calcareous soil developed from a limestone base (Lu et al., 2012). The Guzhou catchment was extremely disturbed by deforestation and cultivation. From 2002 onward, some croplands were abandoned with the introduction of the Grain for Green project (Hu et al., 2018) and were listed as pilot areas for ecological restoration and the reconstruction of depressions in karst hills. We chose seven GfG sites on slopes that were formally cropland (*Zea mays*-*Glycine max*). Four of the sites were afforested using one of the following tree species: *Zenia insignis* (ZI), *Toona sinensis* (TS), *Castanea mollissima* (CM), and *Citrus reticulate* (CR). One site was revegetated with *Zenia insignis* and Guimu-1 elephant grass (ZG), and the other with only Guimu-1 elephant grass (GM). The remaining site was abandoned cropland (AC), in which vegetation regenerated naturally.

We sampled one plot (20 m × 20 m) in December 2017 at each site, and the plots had a similar slope position, degree, and aspect. We removed the litter layer and collected 15 samples in an “S” shape from the top 20 cm of soil using a soil auger (2.5 cm inner diameter), and then homogenized these samples to provide one final soil sample. This sampling method was performed in triplicate at each site, giving us a total of 21 samples. At the same time, we investigated plants in the plots. For arboreal layers, all trees with a diameter at breast height (DBH, or 1.30 m above grade) ≥ 1 cm were tagged, identified, and measured. For the shrubby and herb layers, the fascicles, heights, and growth status of each plant species were recorded. The plant diversities (Species richness, Shannon-Wiener index, Simpson index, and Pielou evenness index) were calculated using the method detailed by Song (2016).

### Soil Chemical Parameters

For soil analysis, each composite sample was divided into two parts. One part was put in a liquid nitrogen tank immediately and stored at −80°C for DNA extraction after sieving and removing debris thoroughly. Another was packed temporarily. All soil samples were then sent to the lab. The remaining samples were sieved through 0.15-mm and 1-mm mesh, air-dried, and stored at ambient air temperature before chemical analysis for total and available elements. We determined soil pH, organic carbon (SOC), total nitrogen (TN), total phosphorus (TP), total potassium (TK), available nitrogen (AN), available phosphorus (AP), and available potassium (AK) according to methods described by Bao (2000).

### DNA Extraction and Purification

Soil bacterial DNA was extracted from 0.5 g fresh soil with a soil DNA kit (Fast DNASPIN Kit for Soil, MP). The concentration and purity of the extracted DNA were measured by electrophoresis on 1.0% agarose and then stored at −80°C until use. The V3–V4 domain of bacterial 16S rRNA was targeted and amplified via PCR amplification with the primers for bacteria, 338F (5’-ACTCCTACGGGAGGCAGCAG-3’) and 806R (GGACTACHVGGGTWTCTAAT-3’; Biddle et al., 2008). The reaction mixture contained 4 μL 5 × FastPfu buffer, 2 μL 2.5 mM dNTPs, 0.2 μL BSA, 0.8 μL each primer (5 μM), 10 ng template DNA, and H_2_O to a final volume of 20 μL (Liang et al., 2017). The samples were set at 95°C for 3 min to be denatured, and then amplified by 30 cycles of 95°C for 10 s, 55°C for 30 s, and 72°C for 45 s, followed by an extension at 72°C for 10 min. All samples were amplified in three replicates and then the relative amplicons were mixed to one final PCR product. In addition, each mixed gene was detected using electrophoresis detection on 2% agarose gels. Finally, a total of 21 PCR products were achieved and conducted on the Illumina MiSeq platform (PE300, Majorbio Bio-Pharm Technology, Co., Ltd., Shanghai, China).

### Bacterial 16S rRNA Gene Sequencing and Processing

Bacterial raw reads were deposited in the NCBI sequence read archive (SRA) under the submission ID SUB6443418 and BioProject ID PRJNA636983. The reads were processed using QIIME (Caporaso et al., 2010). Partial 16S rRNA bacterial sequences were filtered using Mothur version 1.22.2 (Schloss et al., 2009) using the criteria of mean quality score ≥ 20 and length ≥ 250 bp. Sequences, appointed to samples, were conducted by exact matches of 10 bp barcodes. Then we used the Uchime algorithm to detect chimeric sequences (Edgar et al., 2011) via the Usearch tool. All chimeras were removed before further analysis. Operational taxonomic units (OTUs) were clustered at the 97% similarity level using UPARSE version 7.1 (Edgar, 2010). The representative sequences were searched against Silva (SSU132) database and analyzed by RDP Classifier using confidence threshold > 70% to generate the final OTUs (Wang et al., 2007).

### Statistical Analyses

All data were tested for normal distribution and variance homogeneity before analysis. The taxonomic alpha diversity indices, Shannon index and Simpson index, and species richness indices, Chao1 and abundance-based coverage estimators (ACE), of soil bacterial community were determined using Mothur software (Version v.1.30.1) based on individual samples with 97% similarity of OTUs. The shared and unique OTUs among samples were used to generate Venn diagrams. Significant differences in soil properties among the different sites were tested using the Kruskal–Wallis non-parametric test. Pairwise comparisons were made with the Wilcoxon rank-sum test and the adjusted *p*-value was used to determine significance. Differences in bacterial diversity indices and differential OTUs (relative abundance > 1%) among sites were tested by one-way analysis of variance (ANOVA), and multiple comparisons were made with Duncan’s test and the least significant difference (LSD), respectively. The *p*-value of the latter were adjusted using the Bonferroni method, of which the *p*-value was divided by the number of comparisons (i.e., the statistical significance was determined at *p* < 0.0024). The Pielou index could not be calculated at the GM site, so we only calculated the mean value and standard error of all plant diversity indices. To investigate the differences in the soil bacterial communities of the seven revegetation sites, we used a heatmap analysis of the most abundant genera with total relative abundance > 1%. The bacterial genera data were centered log-ratio (clr) transformed to fulfill normality of residuals. Correlations between the environmental factors and the soil bacterial genera were investigated using Pearson’s correlation analysis, and the correlation among environmental factors were also analyzed. We reported the Pearson correlation values among environmental factors that were over 0.3 and were significant (*p* < 0.05), and reported all Pearson correlation values between environmental factors and soil bacterial genera. We used the R environment (v3.2.2^1^) for the above-mentioned statistical analyses. Redundancy analysis was performed at the genus level. The analysis with Monte Carlo permutations (999 repetitions) was performed with Canoco (version 5.0 for Windows; Ithaca, NY, United States). The RDA was with forward selection to select the explanatory environmental factors that contributed significantly (*p* < 0.05) to the variation of the soil bacterial community.

## Results

### Soil Properties and Plant Diversity in Different Sites

**FIGURE 1:**
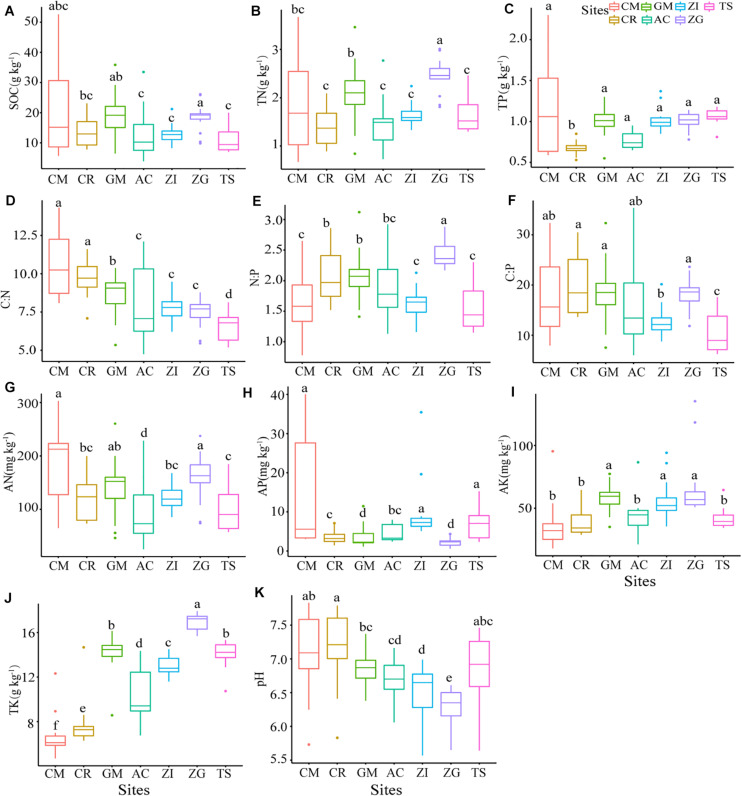
The soil properties at different sites. Boxplots that do not share a letter are significantly different among different sites (*p* < 0.05). ZI, Zenia insignis; TS, Toona sinensis; CM, Castanea mollissima; CR, Citrus reticulate; ZG, Zenia insignis and Guimu-1 elephant grass; GM, Guimu-1 elephant grass; AC, abandoned cropland; SOC, soil organic carbon; TN, total nitrogen; TP, total phosphorus; TK, total potassium; AP, available phosphorus; AK, available potassium; AN, available nitrogen; C:N, C:N ratio; C:P, C:P ratio; N:P, N:P ratio.

All soil properties had significant differences (*p* < 0.05) among the different Grain for Green sites (Figure 1). The median content of SOC varied from 3.90 to 52.58 g.kg^–1^. The SOC content was highest in ZG and lowest in TS. The sites ZI and ZG had a more concentrated distribution of SOC, while CM had the largest variance. The C:N ratio was highest in CM with a large variance, and the ratio was from highest to lowest: CM > CR > GM > ZI > ZG > AC > TS. The C:P ratio and N:P ratio had the same descending order: ZG > GM > CR > AC > ZI > TS. The C:P ratio at TS was significantly lower than at under other sites (*p* < 0.05). The N:P ratio fluctuated gently, ranging from 1.44 to 2.36, compared to the other two ratios. The N:P ratio was significantly higher in ZG that at the other sites (*p* < 0.05). Our study area for the Grain for Green sites had slightly acidic and neutral soil with pH values that ranged from 6.0 to 7.2. The TN and TK contents were highest in ZG. The sites TS and CM had the highest median content of TP compared to the other sites.

The plant species richness (S) and Shannon-Wiener indices were low in all sites, especially in GM site (Table 1), because of the lack of species diversity among the plants used for revegetation. The four indices were all highest in the XC site. The plant species richness and Shannon-Wiener indices, from highest to lowest, were for the TS, ZG, AC, ZI, CM, CR, and GM sites. The plants at each site were distributed evenly with high values of Pielou evenness index (Table 1).

**TABLE 1:** Plant diversity indices for different sites.

	S	Shannon	Simpson	Pielou
ZG	6.00 ± 1.53	1.43 ± 0.22	0.70 ± 0.05	0.83 ± 0.01
ZI	5.33 ± 0.88	1.16 ± 0.09	0.59 ± 0.05	0.71 ± 0.08
CR	3.33 ± 0.88	0.81 ± 0.17	0.48 ± 0.08	0.72 ± 0.02
CM	3.67 ± 0.33	1.00 ± 0.08	0.56 ± 0.03	0.78 ± 0.03
AC	5.67 ± 0.67	1.31 ± 0.18	0.63 ± 0.09	0.76 ± 0.11
TS	6.33 ± 1.20	1.60 ± 0.23	0.76 ± 0.05	0.88 ± 0.03
GM	1.00 ± 0.00	0.00 ± 0.00	0.00 ± 0.00	NA

**Data are mean ± standard error. ZI, Zenia insignis; TS, Toona sinensis; CM, Castanea mollissima; CR, Citrus reticulate; ZG, Zenia insignis and Guimu-1 elephant grass; GM, Guimu-1 elephant grass; AC, abandoned cropland*.*

### Composition and Structure of Soil Bacteria Under Different Approaches

A total of 1,028,285 raw sequences were obtained from the seven sites, and the average length of valid sequences was 457.02. Rarefaction curves for bacterial community at distance levels of 0.03 had reached an asymptote (Supplementary Figure S1), which indicated that the sequence was sufficient to represent the different bacterial communities. Thus, our data were sufficient to allow an analysis of bacterial communities. We illustrated the similarities and differences among the OTUs of the sites in two four-set Venn diagrams (Supplementary Figures S2, S3). The unique OTUs were 51, 48, 54, and 125 for the ZI, ZG, CR, and AC sites, respectively; the four patterns shared 1285 OTUs (Supplementary Figure S2). The unique OTUs were 96, 57, 53, and 63 for the CM, TS, GM, and AC sites, respectively; the four patterns shared 1225 OTUs (Supplementary Figure S3).

**FIGURE 2:**
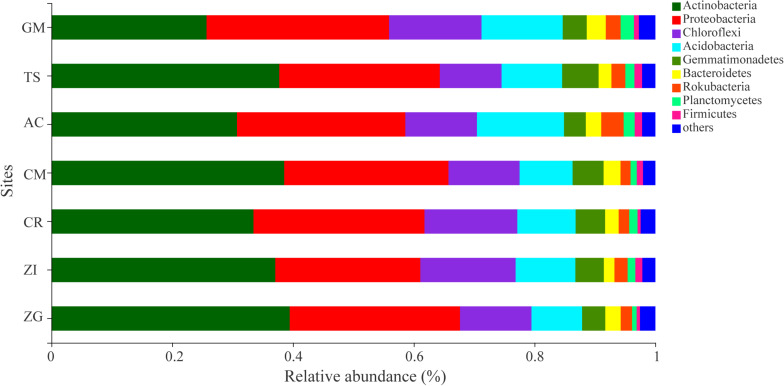
The average relative abundance of soil bacterial phylum. ZI: Zenia insignis; TS: Toona sinensis; CM: Castanea mollissima; CR: Citrus reticulate; ZG: Zenia insignis and Guimu-1 elephant grass; GM: Guimu-1 elephant grass; AC: abandoned cropland.

The dominant bacterial OTUs (relative abundance > 1%) belong to Actinobacteria and Proteobacteria phyla (Supplementary Table S2). These dominant OTUs were assigned to a total of 6 bacterial genus including Micromonospora, Streptomyces, Lysobacter, Micrococcaceae, Bradyrhizobium, Intrasporangiaceae. And a higher relative abundance of Micromonospora (OTU2141, 2139, 2459), Lysobacter (OTU2463), and Streptomyces (OTU2136, 1650) were observed in TS and ZG sites, respectively. The relative abundance of the bacterial taxa at the phylum level varied among the different sites (Figure 2). Actinobacteria, Proteobacteria, Chloroflexi, and Acidobacteria were the dominant bacterial phyla, which occupied 86.0% of the total bacterial abundance (Figure 2). Actinobactria was the most abundant phylum (34.67%) with only one class. Moreover, the relative abundance of Actinobacteria in the ZG, CM, ZI, and AC sites were more than 37%. Proteobacteria was the second most abundant phylum in these revegetated sites, with Gamma- (12.71%) and Alpha-proteobacteria (9.75%) being the most dominant classes next to Actinobacteria (Supplementary Figure S4). At the genus level, the dominant bacterial taxa were Micromonospora, Lysobacter, Subgroup 6, and Streptomyces, which belonged to the Actinobacteria, Proteobacteria, Acidobacteria, and Actinobacteria phyla, respectively (Supplementary Figure S5). The genera in the Chloroflexi phylum division, such as A4b, Roseiflexaceae, SBR1031, and KD4-96, were in medium relative abundance. Micromonospora had the highest average relative abundance and dominated in the AC and XC sites, but was depressed in the CR and GM sites. The Streptomyces (Actinobacteria phylum) and Subgroup 6 (Acidobacteria phylum) was dominant in the ZG and GM sites, respectively.

**FIGURE 3:**
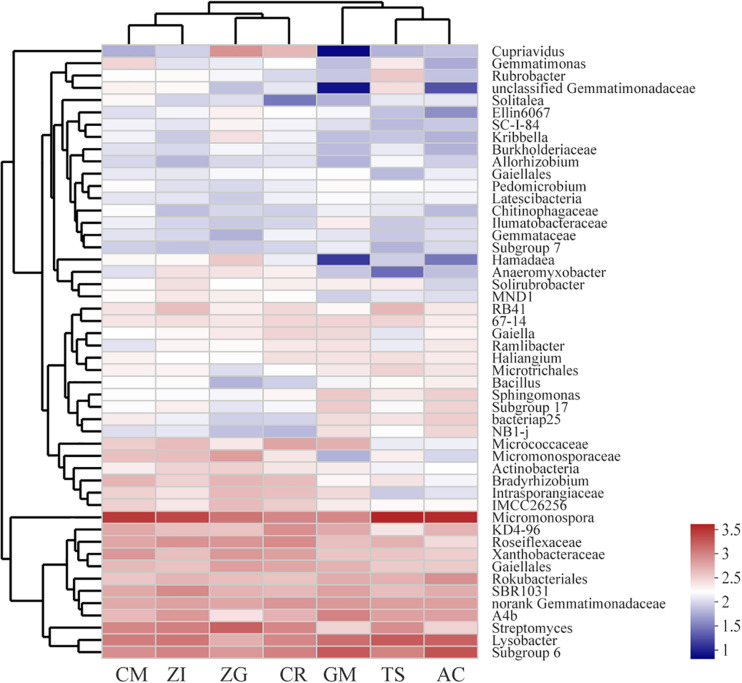
Heatmap and hierarchical cluster analysis based on the relative abundance of the top 50 genera identified in the bacterial communities of the soil. The cluster analysis (average linkage) was performed with soil samples, and similarity was calculated using the Bray–Curtis method. ZI: Zenia insignis; TS: Toona sinensis; CM: Castanea mollissima; CR: Citrus reticulate; ZG: Zenia insignis and Guimu-1 elephant grass; GM: Guimu-1 elephant grass; AC: abandoned cropland.

Our heatmap showed that the bacterial community could be divided into five clusters (Figure 3). The abundances of these bacterial genera were different among the seven revegetation sites. The dominant genera in each revegetation site were also different. For example, the CM, ZI, TS, and AC sites were dominated by Micromonospora, whereas the ZG, CR, and GM sites were enriched by Streptomyces and Subgroup 6. Overall, the Micromonospora almost had the highest abundance among all sites (Supplementary Figure S5). The bacterial composition and distribution were similar in the AC and TS sites. These two sites all had a higher abundance of Micromonospora, Lysobacter, and Subgroup 6. Nevertheless, the GM site had the lowest amount of *Cupriavidus*, unclassified Gemmatimonadaceae, and Hamadaea. The differences in bacterial abundance in the other four sites were not large.

### Diversity of Bacterial Communities Under Different Approaches

The depth of all sample sequences was over 98% (Table 2), which indicated that sequence coverage was adequate and met the criteria of our experiments. The diversity indices for the soil bacterial community varied among the different sites (Table 2). The ACE and Chao1 indices had the same decreasing trend in the order of ZI, CM, GM, CR, AC, TS, and ZG. Site GM had the highest richness of bacteria, followed by CM, GM, CR, AC, TS, and ZG. The Chao1 index was higher in ZI, CM, GM, and CR, followed by AC, TS, and ZG. The ACE index was highest in ZI, and there was no significant differences among all sites. The Shannon-Wiener index was highest in GM and lowest in TS, and there was no significant differences in the index among the other sites. The Simpson index was highest in TS site, which was significantly higher than in GM (*p* < 0.05) site.

**TABLE 2:** Soil bacterial diversity indices of different sites.

	Shannon	Simpson	ACE	Chao1	Coverage
ZG	5.55 ± 0.33ab	0.01 ± 0.00ab	1544.34 ± 141.65	1588.81 ± 143.07	0.99 ± 0.00
ZI	5.87 ± 0.05ab	0.01 ± 0.00ab	1787.63 ± 46.79	1816.77 ± 50.13	0.98 ± 0.00
CR	5.92 ± 0.03ab	0.01 ± 0.00ab	1689.90 ± 108.74	1717.89 ± 99.50	0.99 ± 0.00
CM	5.76 ± 0.03ab	0.01 ± 0.00ab	1766.77 ± 57.96	1785.30 ± 40.39	0.98 ± 0.00
AC	5.61 ± 0.44ab	0.02 ± 0.02ab	1638.50 ± 83.20	1673.24 ± 85.51	0.99 ± 0.00
TS	5.43 ± 0.09b	0.03 ± 0.00a	1615.95 ± 31.97	1615.86 ± 38.70	0.99 ± 0.00
GM	6.21 ± 0.05a	0.01 ± 0.00b	1737.09 ± 58.17	1773.05 ± 49.06	0.99 ± 0.00

**Data are mean ± standard error. Different lowercase letters in the same column indicate significant difference at 0.05 level. ZI, Zenia insignis; TS, Toona sinensis; CM, Castanea mollissima; CR, Citrus reticulate; ZG, Zenia insignis and Guimu-1 elephant grass; GM, Guimu-1 elephant grass; AC, abandoned cropland*.*

### Effects of Environmental Factors on Bacterial Communities

The three soil stoichiometric ratios and plant diversity indices had a significant effect on soil bacterial genera (Table 3). Bacterial Shannon-Wiener index was significantly negatively correlated with plant Shannon-Wiener and Simpson indices (Supplementary Figure S6). The relative abundance of KD4-96 was largely affected by soil properties and plant diversity indices. The ratio of C:P had positive effects on KD4-96, Intrasporangiaceae, and *Gaiella*, and negative effects on RB41 and *Rubrobacter*. The ratio of N:P was significantly positively correlated with *Cupriavidus* and *Kribbella*, and negatively with Microtrichales. Only one genus of Proteobacteria (*Cupriavidus*), Acidobacteria (RB41), and Gemmatimonadetes (unclassified Gemmatimonadaceae) were very significant correlated with soil stoichiometric ratios (*p* < 0.01).

**TABLE 3:** The correlation coefficients between soil bacterial genera and environmental variables.

	pH	SOC	TN	TP	TK	AN	AP	AK	C:N	C:P	N:P	S	Shan	Simp
Micro	0.17	–0.18	–0.19	0.16	–0.05	–0.21	0.17	–0.23	–0.13	–0.37	–0.37	0.32	0.45*	0.49*
Lys	0.04	–0.07	–0.13	0.13	–0.06	–0.18	0.12	–0.16	–0.06	–0.25	–0.33	0.10	–0.01	–0.09
Sub 6	–0.02	0.00	–0.12	–0.16	–0.03	–0.23	–0.10	0.09	0.04	0.10	–0.01	–0.09	–0.19	–0.21
Strep	–0.19	0.03	0.26	0.00	0.18	0.28	–0.11	0.09	–0.12	0.09	0.33	0.20	0.34	0.43
Gem	0.16	0.12	0.10	–0.05	0.04	0.12	–0.14	0.11	0.08	0.24	0.18	–0.07	–0.14	–0.16
A4b	0.15	–0.03	–0.12	0.08	0.02	–0.18	0.10	0.01	0.03	–0.11	–0.27	–0.10	–0.30	–0.39
Rose	0.02	–0.06	–0.03	–0.13	–0.07	0.08	–0.08	0.15	0.08	0.14	0.16	–0.02	0.11	0.20
SBR	–0.20	–0.19	–0.18	–0.10	–0.02	–0.09	–0.01	0.01	–0.12	–0.21	–0.21	–0.04	–0.15	–0.16
Xan	–0.01	0.22	0.10	0.01	–0.29	0.34	0.12	–0.15	0.32	0.25	0.15	0.02	0.08	0.14
Roku	–0.11	–0.21	–0.20	–0.18	0.02	–0.39	–0.10	–0.02	–0.20	–0.11	–0.08	0.15	–0.00	–0.07
KD4	0.30	0.13	–0.27	–0.42	−0.58**	0.10	–0.10	–0.19	0.60**	0.58**	0.19	−0.48*	−0.50*	−0.46*
Gai	–0.10	0.19	0.27	–0.16	0.10	0.23	–0.23	0.34	0.13	0.43	0.53*	–0.26	–0.26	–0.22
Micoc	0.52*	0.30	0.04	0.11	–0.22	0.24	0.30	–0.02	0.51*	0.36	0.06	–0.39	–0.40	–0.37
Brad	0.04	0.18	0.12	0.05	–0.22	0.39	0.13	–0.23	0.26	0.20	0.13	0.08	0.15	0.20
Mimon	–0.23	0.07	0.27	0.05	0.17	0.30	–0.05	0.09	–0.09	0.03	0.25	0.32	0.42	0.49*
RB41	0.04	–0.34	–0.21	0.13	0.08	–0.22	0.11	–0.10	–0.32	−0.45*	–0.37	0.44*	0.50*	0.52*
Int	–0.10	0.18	0.17	–0.21	–0.04	0.28	–0.19	0.24	0.25	0.44*	0.47*	0.38	–0.32	–0.25
IMC	–0.23	0.21	0.29	–0.15	0.00	0.39	–0.19	0.16	0.15	0.40	0.52*	–0.05	0.02	0.08
67–14	0.17	–0.04	–0.06	0.03	–0.02	–0.13	0.00	–0.05	0.01	–0.03	–0.11	–0.06	–0.11	–0.14
Actin	–0.17	0.01	0.11	–0.07	0.15	0.03	–0.06	0.36	–0.04	0.13	0.25	–0.12	–0.11	–0.06
Sph	–0.07	0.02	0.00	–0.15	0.11	–0.16	–0.26	0.11	–0.06	0.09	0.07	–0.26	–0.36	−0.45*
Cup	–0.16	0.04	0.27	–0.19	0.25	0.13	–0.27	0.34	–0.11	0.30	0.60 **	–0.11	0.21	0.28
Hal	0.00	–0.12	–0.19	–0.17	–0.12	–0.14	–0.14	–0.07	–0.02	0.02	–0.06	–0.20	–0.22	–0.25
Gaie	0.11	0.11	0.00	–0.41	–0.10	0.03	–0.35	0.23	0.28	0.57**	0.48*	−0.53*	−**0.59**	−0.58**
Mitri	0.24	–0.20	–0.29	0.11	–0.14	–0.31	0.14	–0.38	–0.14	–0.37	−0.49*	0.12	0.06	0.00
Sub 17	0.08	0.03	–0.10	–0.02	0.02	–0.23	0.03	0.10	0.04	–0.01	–0.15	–0.25	−0.44*	−0.52*
Bact	0.04	0.03	–0.11	0.10	–0.12	–0.17	0.11	–0.17	–0.03	–0.15	–0.31	–0.03	–0.16	–0.24
Sol	0.08	–0.08	–0.03	0.19	0.14	–0.05	0.15	0.13	–0.05	–0.20	–0.23	–0.13	–0.16	–0.15
Ram	0.01	0.11	0.06	–0.07	0.08	–0.01	–0.06	0.23	0.10	0.24	0.16	–0.03	–0.21	–0.29
Rub	0.02	–0.21	–0.05	0.34	0.16	–0.17	0.24	–0.19	–0.36	−0.53*	–0.42	0.48*	0.48*	0.46*
MN	–0.13	0.10	0.15	0.11	0.04	0.24	0.10	0.29	0.03	0.07	0.12	0.24	0.30	0.36
NB	–0.06	0.01	–0.04	–0.01	0.07	–0.21	–0.06	0.07	–0.08	–0.06	–0.13	–0.08	–0.24	–0.34
Gema	0.14	0.12	–0.05	0.18	–0.38	0.32	0.20	–0.38	0.23	–0.05	–0.27	0.09	0.21	0.28
Ana	–0.17	0.02	0.14	–0.12	0.10	0.25	–0.10	0.18	–0.02	0.16	0.31	0.03	0.09	0.14
Ham	–0.26	0.04	0.26	–0.01	0.17	0.27	–0.09	0.10	–0.11	0.08	0.32	0.19	0.28	0.35
Gemat	0.06	–0.16	–0.16	0.25	–0.10	–0.02	0.28	–0.24	–0.12	–0.40	−0.45*	0.40	0.46*	0.50*
Kri	–0.16	0.17	0.36	–0.07	0.17	0.32	–0.20	0.18	0.00	0.31	0.54*	0.00	0.14	0.22

*Indicated that correlation was significant at the 0.05 level (two-tailed). **indicated that correlation was significant at the 0.01 level (two-tailed). The bold values meant that the correlation values were significant at 0.05 or 0.01 level. SOC, soil organic carbon; TN, total nitrogen; TP, total phosphorus; TK, total potassium; AP, available phosphorus; AK, available potassium; AN, available nitrogen; C:N, C:N ratio; C:P, C:P ratio; N:P, N:P ratio; S, Species richness; Shan, Shannon-Wiener index; Simp, Simpson index; Micro, Micromonospora; Lys, Lysobacter; Sub 6, Subgroup 6; A4b, A4b; Rose, Roseiflexaceae; Gem, norank Gemmatimonadaceae; SBR, SBR1031; Xan, Xanthobacteraceae; Roku, Rokubacteriales; KD4, KD4-96; Kri, Kribbella; Gai, Gaiellales; Micoc, Micrococcaceae; Brad, Bradyrhizobium; Mimon, Micromonosporaceae; Int, Intrasporangiaceae; IMC, IMCC26256; Actin, Actinobacteria; Mitri, Microtrichales; Sub 17, Subgroup 17; Bact, bacteriap25; NB, NB1-j; Gema, Gemmatimonas; Strep, Streptomyces; Sph, Sphingomonas; Cup, Cupriavidus; Hal, Haliangium; Micro, Microtrichales; Sol, Solirubrobacter; Ram, Ramlibacter; Rub, Rubrobacter; MN, MND1; Gaie, Gaiella; Ana, Anaeromyxobacter; Ham, Hamadaea; Gemat, unclassified Gemmatimonadaceae.

**FIGURE 4:**
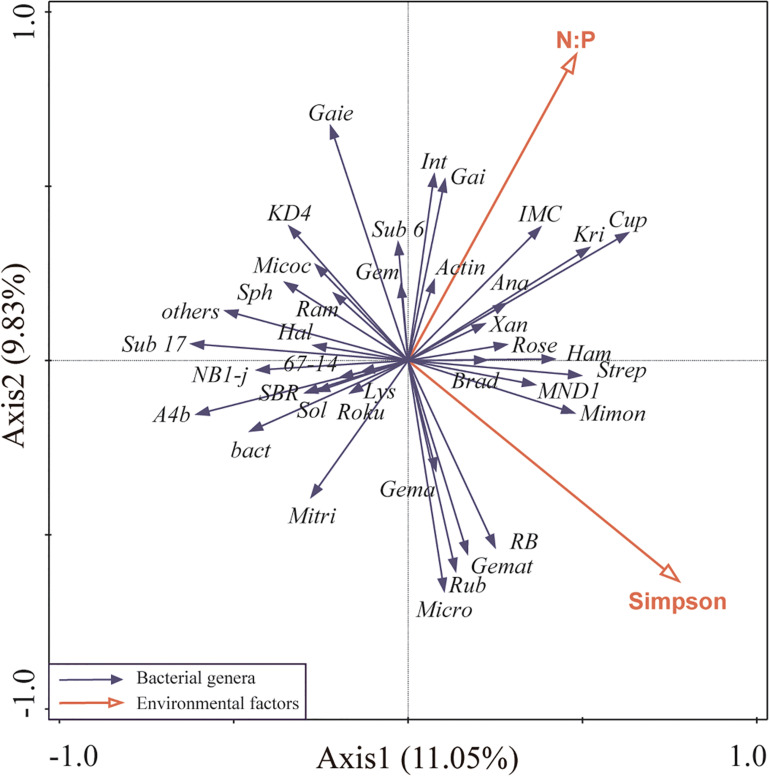
Ordination plots of the redundancy analysis (RDA) to identify the relationship between the abundance of bacterial taxa (black arrows) and environmental variables (red arrows). Plant Shannon index was highly correlated with plant Simpson index and species richness (Pearson R = 0.97, R = 0.92, respectively), thus was removed from this analysis. These are the abbreviations of environmental variables and bacterial taxa: N:P, soil N:P ratio; Simpson, plant Simpson index; Micro, Micromonospora; Lys, Lysobacter; Sub 6, Subgroup 6; A4b, A4b; Rose, Roseiflexaceae; Gem, norank Gemmatimonadaceae; SBR, SBR1031; Xan, Xanthobacteraceae; Roku, Rokubacteriales; KD4, KD4-96; Kri, Kribbella; Gai, Gaiellales; Micoc, Micrococcaceae; Brad, Bradyrhizobium; Mimon, Micromonosporaceae; Int, Intrasporangiaceae; IMC, IMCC26256; Actin, Actinobacteria; Mitri, Microtrichales; Sub 17, Subgroup 17; bact, bacteriap25; Gema, Gemmatimonas; Strep, Streptomyces; RB, RB41; Sph, Sphingomonas; Cup, Cupriavidus; Hal, Haliangium; Micro, Microtrichales; Sol, Solirubrobacter; Ram, Ramlibacter; Rub, Rubrobacter; Ana, Anaeromyxobacter; Ham, Hamadaea; Gemat, unclassified Gemmatimonadaceae; Gaiella, Gaie.

For highly correlation of plant Shannon index with other environmental factors, it was removed from RDA analysis. Redundancy analysis of the main bacterial genera showed that the first two axes of the redundancy analysis accounted for 20.88% of the total variance in the bacterial community composition, with the first axis accounting for 11.05% of the variance (Figure 4). Plant Simpson index, and soil N:P were more correlated to bacterial community composition than other environmental factors, contributing 16% (*P* = 0.05), and 15.7% (*P* = 0.04), respectively, which suggested that they were the main factors shaping soil bacterial community (Figure 4).

## Discussion

### Soil Properties Under Revegetation

The GfG program used afforestation and revegetation as tools for ecological restoration, but the program also aimed to alleviate poverty and hasten rural development. Farming in Guzhou Village intensified soil erosion, and the soil was nutrient deficient and characteristic of the thin, nutrient-poor soils of undisturbed karst systems (Green et al., 2019). Zhang et al. (2019) found that vegetation restoration (e.g., natural restoration, artificial forests, and artificial grassland) significantly improved soil quality, which was confirmed in our study. We also found that plant richness was significantly negatively correlated with the ratio of C:N and C:P (Supplementary Figure S6), which indicated higher plant diversity might contribute to decreasing nutrient limitation.

Soil organic matter plays a key role in soil function, soil quality, water holding capacity, and the susceptibility of soil to degradation (Fragoso et al., 1997). The soil at our study sites had a relatively lower SOC content (15.66 ± 8.41 g.kg^–1^, data not shown) than what was found at other sites (81.12 ± 15.89 g.kg^–1^, 21.07–77.13 g.kg^–1^; Liu et al., 2016; Zhu et al., 2017). The difference might be because of the distinctive geology and climate, and the leaching of solubilized microelements from the soil (Beynen, 2011). Due to intensive soil erosion via water in the sub-tropical karst ecosystem, SOC loss was accelerated (Green et al., 2019) and complicated afforestation efforts. The content of humic acid in soil has been found to be positively correlated with organic matter content (Wu et al., 2019), which we also found in our study in that the soils with higher SOC had lower pH in ZG, ZI, AC, and TS sites (Figure 1). However, SOC and pH did not show a strong correlation (Supplementary Figure S6), perhaps because pH was near neutral.

Feng et al. (2018) pointed out that soil nitrogen availability was a major constraint for plant growth and consequently impacted soil carbon sequestration following afforestation, and we found that SOC content was significantly positively correlated with AN and TN (Supplementary Figure S6). This finding indicated that our soil nitrogen might constrain soil carbon storage by affecting the productivity of plants. The contents of other soil properties and stoichiometric ratios were not high, especially the parameters related to phosphorus, which further indicated that the soils were phosphorus deficient. During the latter period of the GfG program, the values of SOC, TN, TK, AN, AK, C:P, and N:P at the ZI and GM sites were less than at ZG (Figure 1), which indicated that the soil nutrient accumulation of revegetation with both tree and grass species on soil was prior than that of single-planted trees or single-planted pastures (Han, 2017). Furthermore, the contents of soil properties and the stoichiometric ratios in the ZG site were almost the highest, which indicated that the ZG approach had the best effects on soil nutrients accumulation. The SOC was lowest in the TS site, which indicated that planting *Toona sinensis* is not very conducive of SOC accumulation. Due to fertilization and other human management in our study area during the early stages of the GfG program, plant residues decomposed rapidly into the soil and the alkaline substances in the soil were neutralized (Han, 2017). The reduction human interference during the mid-stages of the project caused the soils to be slightly acidic or neutral at all sites (Figure 1).

### Soil Bacterial Community Under Revegetation

Actinobacteria, Proteobacteria, Chloroflexi, and Acidobacteria were the dominant phyla at all sites (Figure 2), and similar results were observed in soils in different environments (Eo and Park, 2016; Xue et al., 2016). Proteobacteria and Actinobacteria were promoted by soil organic carbon, while Acidobacteria, an oligotrophs (Hartman et al., 2008), was suppressed by soil organic carbon (Zhou et al., 2017). Even Acidobacteria thrived in soils with low resource availability, which was widespread in soil (Fierer et al., 2007). Therefore, every site had Acidobacteria (Figure 2). Soil was neutral or slightly acidic, which was more suitable for the survival of Streptomyces (Actinobacteria phylum). Proteobacteria and Bacteroidetes were carbon- and nitrite-oxidizing bacterial group with higher relative abundance in all sites, which indicated that these bacterial groups had positive effects on the fertility of soil under the revegetation approaches.

Due to its multi-layer canopy structure, the combination of *Zenia insignis* and Guimu-1 elephant grass site had the best edaphic benefits (Figure 1). Although *Zenia insignis* grows rapidly and consumes a lot of nutrients, Guimu-1 elephant grass created an abundant source of SOC and nutrients (Han, 2017). A more diverse plant community could also provide more diverse ecological niches that increase the growth and abundance of soil bacteria. The soil at the CM site had the lowest concentration of potassium (Figure 1) and the highest abundance of Actinobacteria (Figure 2), which might be because the CM site had the highest C:N ratio to meet the need of Actinobacteria growth. The *Toona sinensis* site had median edaphic property contents and stoichiometric ratios (Figure 1) and a higher relative abundance of dominant bacteria (Figure 3 and Supplementary Table S1), which might have been due to its high total phosphorus as a source for AP transformation. The variation of relative abundance among the different bacterial phyla (Figure 2) at the GM site, which consumed a large amount of phosphorus and yielded vigorous plant growth (Han, 2017), was smaller than other sites. Thus, when planting Guimu-1 elephant grass, phosphate fertilizer was needed. The *Zenia insignis* site had relatively low soil nutrients (Figure 1) due to the consumption of a considerable amount of phosphorus (Lu et al., 2012) and necessitated soil fertilization management. The *Citrus reticulate* and abandoned cropland sites had the worst edaphic effect (Figure 1), and the introduction of other plant species was considered to hasten ecological restoration.

The bacterial community structure differed with vegetation, which indicated that the plant species chosen for afforestation and revegetation were good indicators of soil bacterial community structure. The bacterial composition and distribution were similar in the AC and TS sites (Figure 3), and they all had the highest abundance of same OTUs (OTU2141, 2463, 649, 219 et al.). This similarity could have been due to the two sites having the most proximate values of soil properties and C:N, such as SOC, TN, and AK. Compared to the monoculture GM site, the plants in the AC site were a mixture of trees and grasses, thus the plant-soil environment was closer to that in the TS site. Another reason might be that they had similar species of grasses and a similar plant root environment. FCPU426 and Fibrobacteres were only found at the ZG and CR sites, where there might be some special plant root exudates that attracted these bacteria. The *Toona sinensis* site had the lowest SOC and soil stoichiometric ratios, which might be because they preferred fertile soil. However, our soil was not fertile originally, which affected their subsequent growth and litter quantity. Therefore, monocultures of *Toona sinensis* needs to be improved.

### Effects of Revegetation on Soil Bacterial Community

Proteobacteria and Actinobacteria were the most abundant phyla in soil bacterial communities and were powerful competitors in carbon utilization (Fierer et al., 2007; Ganz et al., 2012), exhibiting rapid growth in carbon-enriched soil (Goldfarb et al., 2011). The abundance of Proteobacteria and Actinobacteria indicated that all revegetation methods in our study had relatively enough soil carbon. At the class level, Actinobacteria was the most abundant (Supplementary Figure S4), and their competitive advantage was evident through their production of antibiotics (D’Costa et al., 2007) and a multitude of degradative capabilities (Zhou et al., 2017). With the accumulation of soil organic matter and nutrients, the relative abundance of Actinobacteria decreased and Proteobacteria increased (Figures 1, 2), which may be caused by the increase in available nutrients (Zeng et al., 2017).

Significant relationships of N:P and C:P with bacterial genera except most dominant genera were observed, which indicated that the variables associated with phosphorus availability may be crucial determinants of bacterial community structure, and the dominant bacterial taxa had the preference of phosphorus. Plant diversity had significant positive effects on some soil bacterial genera, especially the most abundant bacterial genus, Micromonospora, which indicated that revegetation with an abundance of plant species could improve the growth of soil bacteria (Figure 4). The tight connection between soil bacterial genera and plant diversity indices indicated that plant diversity participated in shaping the soil bacterial community (Table 3). The bacterial Shannon–Wiener index was significantly negatively correlated with plant Shannon–Wiener and Simpson indices (Supplementary Figure S6), which indicated that higher plant diversity does not necessarily promote soil bacterial diversity at the intermediate stage of revegetation. An imbalanced fertilization experiment found the percentage of Acidobacteria abundance had a positive correlation with potassium and negative correlation with calcium (Eo and Park, 2016), but we did not find the same correlations in our study (Table 3). The reason might be that high calcium in karst region suppressed the uptake of potassium by Acidobacteria taxa. They also found the percentages of Chloroflexi were positively correlated with pH and negatively correlated with nitrate concentration (Eo and Park, 2016), but we did not observe these correlations (Table 3). The possible reasons for this difference could be that our soil was lacking nitrates and a high pH may suppress the growth of Chloroflexi (Deng et al., 2018). Firmicutes was found to be more adaptable to dry and cold soil environments and used limited soil nutrients (Zhu et al., 2017), while under a humid subtropical climate the abundance of Firmicutes was lower (Figure 2). Zhu et al. (2017) also found that Gemmatimonadetes was more suitable for living in low soil water content environment. Thus, it is likely that the sites AC, ZG, and GM had the lowest abundance of Gemmatimonadetes due to their deep soil and potential to hold water. The Bacillus class, the predominant bacteria for their phosphate-solubilizing abilities, had low abundance in our soil (Supplementary Figure S3) and further indicated that our soils were deficient in phosphorus (Figure 2).

Many studies demonstrated that soil pH was the factor driving soil bacterial community structure (Lauber et al., 2009; Kaiser et al., 2016). However, we found a weak correlation between soil pH and bacterial distribution in our study, which could be due to the relatively wide growth tolerances exhibited by most bacterial taxa. Hu et al. (2016) also found that the karst microorganisms were possibly very tolerant of the poor soil habitat. Although pH correlated well with bacterial community change (Williams et al., 2013), it was not well correlated with similar bacterial community changes when pH was uniform (Jangid et al., 2013). Another reason might be the homogenic soil pH with narrow range of variation (6.0–7.2) in our study area. Combined with nutrient limitation, our bacterial community was affected by the dominance of competitive species and competitive exclusion (Kaiser et al., 2016). Liang et.al (2017) investigated bacterial communities in soils in a karst rocky desertified area in Yunnan, and they found that soil pH, Ca^2+^ content, organic carbon, total nitrogen, and soil moisture jointly influenced bacterial community structure. Their lower pH value might due to acidification caused by coniferous afforestation, and their higher SOC content was due to the input of forest litter. There was more Acidobacteria than in the soils we studied due to acidity, and soil bacteria phyla in our study were in accordance with the previous findings with no significant differences, except for Betaproteobacteria and Armatimonadetes.

Bacterial community structure and diversity were influenced by a range of physicochemical properties (Hu et al., 2016; Sui et al., 2019). All the bacterial diversity indices in a mountainous region (Deng et al., 2018) were higher than those in our study, which might be due to the soil in our study being nutrient limited. Correlation analysis between environmental factors and soil bacterial genera indicated that variables associated with nitrogen and phosphorus transformations may be crucial determinants of bacterial community (Shi et al., 2015), especially phosphorus content (Table 3). The ZG site had the highest soil C:P, lowest AP, and low bacterial diversity, which indicated that soils with low phosphorus and high C:P ratio may constrain bacterial diversity (Delgado-Baquerizo et al., 2017)., and phosphorus availability affected the quantity of N within ecosystems by affecting N fixation (Vitousek et al., 2010). Soil microbial species were usually abundant in habitats with sufficient carbon sources, but our soil bacteria had low diversity when SOC was relatively high, which suggested that the bacterial communities in our soil were constrained by other nutrients, such as nitrogen and phosphorus. We found soil N:P and plant Simpson index jointly influenced bacterial community structure (Figure 4), which indicated that our bacteria community was affected by soil nutrient limitation and plant diversity. In addition, plants had a greater effect on soil bacterial community than soil nutrients. Though revegetation increased SOC content, soil fertility was not adequate. Moreover, the deficiency of nitrogen and phosphorus was characteristic of karst soils in a subtropical climate. Thus, the low nitrogen and phosphorus became the more important limited factors. Fierer and Jackson (2006) and Lauber et al. (2009) described a peak of soil bacterial diversity in near neutral soils. Our soils were near neutral but were limited by soil nutrients, which indicated that soil bacterial diversity may not have peaked. Compared to phosphorus, which was supplied only from parent material, the carbon and nitrogen contents were relatively high enough due to recharging via photosynthesis and nitrogen deposition. Additionally, the massive demand for phosphorus by fast-growing bacteria for the synthesis of ribosomal RNA (Ren et al., 2016) further aggravated phosphorus deficiency. A study in a prairie suggested that increasing plant community richness significantly altered soil bacterial community composition and was negatively correlated with bacterial diversity (Schlatter et al., 2015). The unexplained part of our study may be related to plant productivity and other edaphic properties such as soil matrix, including pore size, particle size, and the availability of water and carbon (Zhu et al., 2017; Song et al., 2018).

## Conclusion

We investigated soil bacterial community structure and diversity at seven revegetated sites in a typical karst rocky ecosystem in southwest China that were part of the GfG program. Our results indicated that the different afforestation and revegetation approaches had different impacts on soil properties and bacterial communities. Actinobacteria, Proteobacteria, Chloroflexi, and Acidobacteria, were the dominant bacterial phyla and occupied 86.0% of the total bacterial abundance. Actinobacteria was the most abundant phylum (34.67%) with only one class. The AC and TS sites had similar bacterial structure. The dominant genus in each revegetation site was also different. The CM, ZI, TS, and AC sites were dominated by Micromonospora, and the ZG, CR and GM sites were dominated by Streptomyces and Subgroup 6. Soil stoichiometric ratio (N:P) and plant Simpson index were the important factors shaping the structure of the bacterial community, and phosphorus was the factor limiting the bacterial community in the GfG program areas in this region. From soil properties accumulation and dominant bacterial genera, we found that most managed vegetation restorations were better than natural vegetation restoration (abandoned cropland) in our study area. The combination of planting *Zenia insignis* and Guimu-1 elephant grass had the best edaphic benefits, and the approach of planting *Citrus reticulate* and *Toona sinensis* needs to be improved. Ultimately, to better improve the outcomes of afforestation and revegetation efforts in the area, soils should be fertilized with phosphorus and polycultures should be considered.

## Data Availability Statement

The datasets generated in this study can be found in online repositories. The names of the repository/repositories and accession number(s) can be found below: https://www.ncbi.nlm.nih.gov/, PRJNA636983.

## Author Contributions

WP and TS conceived the experiments. HC and FW conducted the experiments. HC analyzed the results and wrote the manuscript, which was edited by WP, TS, FZ, and HZ. HD conducted the field sampling. All authors contributed to the article and approved the submitted version.

## Conflict of Interest

The authors declare that the research was conducted in the absence of any commercial or financial relationships that could be construed as a potential conflict of interest.

## Footnotes

1 http://www.r-project.org/

## References

[B1] Bao S. (2000). *Soil and Agricultural Chemistry Analysis.* Beijing: China Agriculture Press.

[B2] Beynen P. E. V. (2011). *Karst Management.* Berlin: Springer Science+Business Media.

[B3] Biddle J. F. Fitzgibbon S. Schuster S. C. Brenchley J. E. House C. H. (2008). Metagenomic signatures of the Peru Margin subseafloor biosphere show a genetically distinct environment. *Proc. Natl. Acad. Sci. U.S.A.* 105 10583–10588. 10.1073/pnas.0709942105 18650394 PMC2492506

[B4] Bonner M. T. L. Allen D. E. Brackin R. Smith T. E. Lewis T. Shoo L. P. (2020). Tropical rainforest restoration plantations are slow to restore the soil biological and organic carbon characteristics of old growth rainforest. *Microb. Ecol.* 79 432–442. 10.1007/s00248-019-01414-7 31372686 PMC7033081

[B5] Caporaso J. G. Kuczynski J. Stombaugh J. Bittinger K. Bushman F. D. Costello E. K. (2010). QIIME allows analysis of high-throughput community sequencing data. *Nat. Methods* 7 335–336. 10.1038/nmeth.f.303 20383131 PMC3156573

[B6] D’Costa V. M. Griffiths E. Wright G. D. (2007). Expanding the soil antibiotic resistome: exploring environmental diversity. *Curr. Opin. Microbiol.* 10 481–489. 10.1016/j.mib.2007.08.009 17951101

[B7] Delgado-Baquerizo M. Reich P. B. Khachane A. N. Campbell C. D. Thomas N. Freitag T. E. (2017). It is elemental: soil nutrient stoichiometry drives bacterial diversity. *Environ. Microbiol.* 19 1176–1188. 10.1111/1462-2920.13642 27943556

[B8] Deng J. Yin Y. Zhu W. Zhou Y. (2018). Variations in soil bacterial community diversity and structures among different revegetation types in the Baishilazi Nature Reserve. *Front. Microbiol.* 9:2874. 10.3389/fmicb.2018.02874 30538689 PMC6277578

[B9] Du H. Peng W. Song T. Wang K. Zeng F. Lu S. (2013). Plant community characteristics and its coupling relationships with soil in depressions between karst hills, North Guangxi, China. *Chin. J. Plant Ecol.* 37 197–208. 10.3724/sp.j.1258.2013.00020

[B10] Edgar R. C. (2010). Search and clustering orders of magnitude faster than BLAST. *Bioinformatics* 26:2460–2461. 10.1093/bioinformatics/btq461 20709691

[B11] Edgar R. C. Haas B. J. Clemente J. C. Quince C. Knight R. (2011). UCHIME improves sensitivity and speed of chimera detection. *Bioinformatics* 27 2194–2200. 10.1093/bioinformatics/btr381 21700674 PMC3150044

[B12] Eo J. Park K.-C. (2016). Long-term effects of imbalanced fertilization on the composition and diversity of soil bacterial community. *Agric. Ecosyst. Environ.* 231 176–182. 10.1016/j.agee.2016.06.039

[B13] Feng J. Wu J. Zhang Q. Zhang D. Li Q. Long C. (2018). Stimulation of nitrogen-hydrolyzing enzymes in soil aggregates mitigates nitrogen constraint for carbon sequestration following afforestation in subtropical China. *Soil Biol. Biochem.* 123 136–144. 10.1016/j.soilbio.2018.05.013

[B14] Fierer N. Bradford M. A. Jackson R. B. (2007). Toward and ecological classification of soil bacteria. *Ecology* 88 1354–1364. 10.1890/05-1839 17601128

[B15] Fierer N. Jackson R. B. (2006). The diversity and biogeography of soil bacterial communities. *Proc. Natl. Acad. Sci. U.S.A.* 103 626–631. 10.1073/pnas.0507535103 16407148 PMC1334650

[B16] Fragoso C. Brown G. G. Patrón J. C. Blanchart E. Lavelle P. Pashanasi B. (1997). Agricultural intensification, soil biodiversity and agroecosystem function in the tropics: the role of earthworms. *Appl. Soil Ecol.* 6 17–35. 10.1016/S0929-1393(96)00154-0

[B17] Ganz H. H. Karaoz U. Getz W. M. Versfeld W. Brodie E. L. (2012). Diversity and structure of soil bacterial communities associated with vultures in an African savanna. *Ecosphere* 3 1–18.

[B18] Goldfarb K. C. Karaoz U. Hanson C. A. Santee C. A. Bradford M. A. Treseder K. K. (2011). Differential growth responses of soil bacterial taxa to carbon substrates of varying chemical recalcitrance. *Front. Microbiol.* 2:94. 10.3389/fmicb.2011.00094 21833332 PMC3153052

[B19] Green S. M. Dungait J. A. J. Tu C. Buss H. L. Sanderson N. Hawkes S. J. (2019). Soil functions and ecosystem services research in the Chinese karst Critical Zone. *Chem. Geol.* 527:119107 10.1016/j.chemgeo.2019.03.018

[B20] Han C. (2017). *Dynamic Change of Ecological Effects of Different Models of Returning Farmland to Forest and Grassland in Karst Peak Cluster Depression.* Changsha: Hunan Agricultural University.

[B21] Hartman W. H. Richardson C. J. Vilgalys R. Bruland G. L. (2008). Environmental and anthropogenic controls over bacterial communities in wetland soils. *Proc. Natl. Acad. Sci. U.S.A.* 105 17842–17847. 10.1073/pnas.0808254105 19004771 PMC2584698

[B22] Herzog S. Wemheuer F. Wemheuer B. Daniel R. (2015). Effects of fertilization and sampling time on composition and diversity of entire and active bacterial communities in German grassland soils. *PLoS One* 10:e0145575. 10.1371/journal.pone.0145575 26694644 PMC4687936

[B23] Hu N. Li H. Tang Z. Li Z. Li G. (2016). Community size, activity and C:N stoichiometry of soil microorganisms following reforestation in a Karst region. *Eur. J. Soil Biol.* 73 77–83. 10.1016/j.ejsobi.2016.01.007

[B24] Hu P. Liu S. Ye Y. Zhang W. He X. Su Y. (2018). Soil carbon and nitrogen accumulation following agricultural abandonment in a subtropical karst region. *Appl. Soil Ecol.* 132 169–178. 10.1016/j.apsoil.2018.09.003

[B25] Jangid K. Whitman W. B. Condron L. M. Turner B. L. Williams M. A. (2013). Soil bacterial community succession during long-term ecosystem development. *Mol. Ecol.* 22 3415–3424. 10.1111/mec.12325 24624422

[B26] Kaiser K. Wemheuer B. Korolkow V. Wemheuer F. Nacke H. Schöning I. (2016). Driving forces of soil bacterial community structure, diversity, and function in temperate grasslands and forests. *Sci. Rep.* 6:33696. 10.1038/srep33696 PMC5030646 27650273

[B27] Lauber C. L. Hamady M. Knight R. Fierer N. (2009). Pyrosequencing-based assessment of soil pH as a predictor of soil bacterial community structure at the continental scale. *Appl. Environ. Microbiol.* 75 5111–5120. 10.1128/aem.00335-09 19502440 PMC2725504

[B28] Liang X. Huadong R. Sheng L. Xiuhui L. Xiaohua Y. (2017). Soil bacterial community structure and co-occurrence pattern during vegetation restoration in karst rocky desertification area. *Front. Microbiol.* 8:2377. 10.3389/fmicb.2017.02377 29250053 PMC5717032

[B29] Liu M. Han G. Zhang Q. (2020). Effects of agricultural abandonment on soil aggregation, soil organic carbon storage and stabilization: results from observation in a small karst catchment, Southwest China. *Agric. Ecosyst. Environ.* 288:106719 10.1016/j.agee.2019.106719

[B30] Liu X. X. Hui C. L. Bi L. Z. Romantschuk M. Kontro M. Strommer R. (2016). Bacterial community structure in atrazine treated reforested farmland in Wuying China. *Appl. Soil Ecol.* 98 39–46. 10.1016/j.apsoil.2015.09.005

[B31] Lu S. Peng W. Song T. Zeng F. Du H. Kl W. (2012). Soil microbial properties under different grain-for-green patterns in depressions between karst hills. *Acta Ecol. Sinica* 32 2390–2399. 10.5846/stxb201103070271

[B32] Pan Y. Cassman N. de Hollander M. Mendes L. W. Korevaar H. Geerts R. H. E. M. (2014). Impact of long-term N, P, K, and NPK fertilization on the composition and potential functions of the bacterial community in grassland soil. *FEMS Microbiol. Ecol.* 90 195–205. 10.1111/1574-6941.12384 25046442

[B33] Peng W. Wang K. Song T. Zeng F. Wang J. (2008). Controlling and restoration models of complex degradation of vulnerable Karst ecosystem. *Acta Ecol. Sinica* 28 811–820.

[B34] Peng W. Zhu Y. Song M. Du H. Song T. Zeng F. (2019). The spatial distribution and drivers of soil microbial richness and diversity in a karst broadleaf forest. *For. Ecol. Manage.* 449:117241 10.1016/j.foreco.2019.03.033

[B35] Ranjard L. Dequiedt S. Jolivet C. Saby N. P. A. Thioulouse J. Harmand J. (2010). Biogeography of soil microbial communities: a review and a description of the ongoing french national initiative. *Agron. Sustain. Dev.* 30 359–365. 10.1051/agro/2009033

[B36] Ren C. Zhang W. Zhong Z. Han X. Yang G. Feng Y. (2017). Differential responses of soil microbial biomass, diversity, and compositions to altitudinal gradients depend on plant and soil characteristics. *Sci. Total Environ.* 610–611 750–758. 10.1016/j.scitotenv.2017.08.110 28822942

[B37] Ren C. Zhao F. Kang D. Yang G. Han X. Tong X. (2016). Linkages of C:N:P stoichiometry and bacterial community in soil following afforestation of former farmland. *For. Ecol. Manage.* 376 59–66. 10.1016/j.foreco.2016.06.004

[B38] Schlatter D. C. Bakker M. G. Bradeen J. M. Kinkel L. L. (2015). Plant community richness and microbial interactions structure bacterial communities in soil. *Ecology* 96 134–142. 10.1890/13-1648.1 26236898

[B39] Schloss P. D. Westcott S. L. Ryabin T. Hall J. R. Hartmann M. Hollister E. B. (2009). Introducing mothur: open-source, platform-independent, community-supported software for describing and comparing microbial communities. *Appl. Environ. Microbiol.* 75 7537–7541. 10.1128/aem.01541-09 19801464 PMC2786419

[B40] Shao P. Liang C. Lynch L. Xie H. Bao X. (2019). Reforestation accelerates soil organic carbon accumulation: evidence from microbial biomarkers. *Soil Biol. Biochem.* 131 182–190. 10.1016/j.soilbio.2019.01.012

[B41] Shi Y. Xiang X. Shen C. Chu H. Neufeld J. D. Walker V. K. (2015). Vegetation-associated impacts on arctic tundra bacterial and microeukaryotic communities. *Appl. Environ. Microbiol.* 81 492–501. 10.1128/aem.03229-14 25362064 PMC4277566

[B42] Song M. Wanxia P. Fuping Z. Hu D. Qin P. Qingguo X. (2018). Spatial patterns and drivers of microbial taxa in a karst broadleaf forest. *Front. Microbiol.* 9:1691. 10.3389/fmicb.2018.01691 30093895 PMC6070632

[B43] Song Y. (2016). *Vegetation Ecology*, 2 Edn Beijing: Higher Education Press.

[B44] Sui X. Zhang R. T. Frey B. Yang L. B. Li M. H. Ni H. W. (2019). Land use change effects on diversity of soil bacterial, acidobacterial and fungal communities in wetlands of the Sanjiang Plain, northeastern China. *Sci. Rep.* 9:18535. 10.1038/s41598-019-55063-4 31811224 PMC6898332

[B45] Tian X. Wang K. Liu Y. Fan H. Wang J. An M. (2020). Effects of polymer materials on soil physicochemical properties and bacterial community structure under drip irrigation. *Appl. Soil Ecol.* 150:103456 10.1016/j.apsoil.2019.103456

[B46] Turley N. E. Bell-Dereske L. Evans S. E. Brudvig L. A. (2019). Agricultural land-use history and restoration impact soil microbial biodiversity. *J. Appl. Ecol.* 57 852–863. 10.1111/1365-2664.13591

[B47] Vitousek P. M. Porder S. Houlton B. Z. Chadwick O. A. (2010). Terrestrial phosphorus limitation: mechanisms, implications, and nitrogen–phosphorus interactions. *Ecol. Appl.* 20 5–15. 10.1890/08-0127.1 20349827

[B48] Wang K. L. Zhang C. H. Chen H. S. Yue Y. M. Zhang W. Zhang M. Y. (2019). Karst landscapes of China: patterns, ecosystem processes and services. *Landsc. Ecol.* 34 2743–2763. 10.1007/s10980-019-00912-w

[B49] Wang Q. Garrity G. Tiedje J. Cole’s J. R. (2007). Naïve Bayesian classifier for rapid assignment of rRNA sequences into the new bacterial taxonomy. *Appl. Environ. Microbiol.* 73 5261–5267. 10.1128/AEM.00062-07 17586664 PMC1950982

[B50] Williams M. A. Jangid K. Shanmugam S. G. Whitman W. B. (2013). Bacterial communities in soil mimic patterns of vegetative succession and ecosystem climax but are resilient to change between seasons. *Soil Biol. Biochem.* 57 749–757. 10.1016/j.soilbio.2012.08.023

[B51] Wu Q. Long J. Li J. Liao H. Liu L. Wu J. (2019). Effects of different microhabitat types on soil microbial community composition in the Maolan Karst Forest in Southwest China. *Acta Ecol. Sinica* 39 1009–1018.

[B52] Xue S. Zhang C. Wang G. Xue S. (2016). Soil bacterial community dynamics reflect changes in plant community and soil properties during the secondary succession of abandoned farmland in the Loess Plateau. *Soil Biol. Biochem.* 97 40–49. 10.1016/j.soilbio.2016.02.013

[B53] Yu Y. Peng W. Song T. Zeng F. Wang K. Wen L. (2014). Stoichiometric characteristics of plant and soil C,N and P in different forest types in depressions between karst hills, Southwest China. *Chin. J. Appl. Ecol.* 25 947–954. 25011284

[B54] Zeng Q. An S. Liu Y. (2017). Soil bacterial community response to vegetation succession after fencing in the grassland of China. *Sci. Total Environ.* 609 2–10. 10.1016/j.scitotenv.2017.07.102 28732294

[B55] Zhang J. Yang X. Song Y. Liu H. Wang G. Xue S. (2020). Revealing the nutrient limitation and cycling for microbes under forest management practices in the Loess Plateau–Ecological stoichiometry. *Geoderma* 361:114108 10.1016/j.geoderma.2019.114108

[B56] Zhang Y. Xu X. Li Z. Liu M. Xu C. Zhang R. (2019). Effects of vegetation restoration on soil quality in degraded karst landscapes of southwest China. *Sci. Total Environ.* 650 2657–2665. 10.1016/j.scitotenv.2018.09.372 30296773

[B57] Zhao F. Ren C. Han X. Yang G. Wang J. Doughty R. (2019). Trends in soil microbial communities in afforestation ecosystem modulated by aggradation phase. *For. Ecol. Manage.* 441 167–175. 10.1016/j.foreco.2019.03.036

[B58] Zhou J. Yang H. Tang F. Koide R. T. Cui M. Liu Y. (2017). Relative roles of competition, environmental selection and spatial processes in structuring soil bacterial communities in the Qinghai-Tibetan Plateau. *Appl. Soil Ecol.* 117–118 223–232. 10.1016/j.apsoil.2017.05.012

[B59] Zhu P. Chen S. Song Y. Han C. Liu G. Chen T. (2017). Soil bacterial community composition and diversity of four representative vegetation types in the middle section of the Qilian Mountains, China. *Acta Ecol. Sinica* 37 3505–3514.

